# Prognostic value of myocardial fibrosis in severe aortic stenosis: study protocol for a prospective observational multi-center study (FIB-AS)

**DOI:** 10.1186/s12872-020-01552-8

**Published:** 2020-06-08

**Authors:** Giedrė Balčiūnaitė, Darius Palionis, Edvardas Žurauskas, Viktor Skorniakov, Vilius Janušauskas, Aleksejus Zorinas, Tomas Zaremba, Nomeda Valevičienė, Audrius Aidietis, Pranas Šerpytis, Kęstutis Ručinskas, Peter Sogaard, Sigita Glaveckaitė

**Affiliations:** 1 Clinic of Cardiac and Vascular Diseases, Institute of Clinical Medicine, Vilnius University Faculty of Medicine, Santariškių str. 2, LT-08661 Vilnius, Lithuania; 2 Department of Radiology, Nuclear Medicine and Medical Physics, Institute of Biomedical Sciences, Vilnius University Faculty of Medicine, Santariškių str. 2, LT-08661 Vilnius, Lithuania; 3 Department of Pathology, Forensic Medicine and Pharmacology, Institute of Biomedical Sciences, Vilnius University Faculty of Medicine, P. Baublio str. 5, LT-08406 Vilnius, Lithuania; 4 Institute of Applied Mathematics, Vilnius University Faculty of Mathematics and Informatics, Naugarduko str. 24, LT-03225 Vilnius, Lithuania; 5 Aalborg University Hospital, Clinical Institute of Aalborg University, Hobrovej 18-22, 9100 Aalborg, Denmark

**Keywords:** Magnetic resonance imaging, Aortic valve stenosis, Myocardial fibrosis, Aortic valve replacement, Prognosis

## Abstract

**Background:**

Adverse cardiac remodeling with a myocardial fibrosis as a key pathophysiologic component may be associated to worse survival in aortic stenosis (AS) patients. Therefore, with the application of advanced cardiac imaging we aim to investigate left ventricular myocardial fibrosis in severe AS patients undergoing aortic valve replacement (AVR) and determine its impact with post-intervention clinical outcomes.

**Methods:**

In a prospective, observational, cohort study patients with severe AS scheduled either for surgical or transcatheter AVR will be recruited from two tertiary heart centers in Denmark and Lithuania. All patients will receive standard of care in accordance with the current guidelines and will undergo additional imaging testing before and after AVR: echocardiography with deformation analysis and cardiovascular magnetic resonance (CMR) with T1 parametric mapping. Those undergoing surgical AVR will also have a myocardial biopsy sampled at the time of a surgery for histological validation. Patients will be recruited over a 2-year period and followed up to 2 years to ascertain clinical outcomes. Follow-up CMR will be performed 12 months following AVR, and echocardiography with deformation analysis will be performed 3, 12, and 24 months following AVR. The study primary outcome is a composite of all-cause mortality and major adverse cardiovascular events.

**Discussion:**

Despite continuous effort of research community there is still a lack of early predictors of left ventricular decompensation in AS, which could improve patient risk stratification and guide the optimal timing for aortic valve intervention, before irreversible left ventricular damage occurs. Advanced cardiac imaging and CMR derived markers of diffuse myocardial fibrosis could be utilized for this purpose. FIB-AS study is intended to invasively and non-invasively assess diffuse myocardial fibrosis in AS patients and investigate its prognostic significance in post-interventional outcomes. The results of the study will expand the current knowledge of cardiac remodeling in AS and will bring additional data on myocardial fibrosis and its clinical implications following AVR.

**Ethics/dissemination:**

The study has full ethical approval and is actively recruiting patients. The results will be disseminated through scientific journals and conference presentations.

**Trial registration:**

ClinicalTrials.govNCT03585933. Registered on 02 July 2018.

## Background

Since increased afterload is the main pathophysiology of aortic stenosis (AS) leading to the progressive development of left ventricular (LV) structural and functional alterations, AS is regarded a disease of the aortic valve, as well as of the myocardium [1]. The increasing wall stress triggers cardiac fibroblasts to upregulate fibronectin synthesis, with subsequent alteration in collagen architecture and myocardial fibrosis, compromising systolic and diastolic LV function [2]. Two distinct types of myocardial fibrosis have been described: (i) diffuse myocardial fibrosis and (ii) focal replacement fibrosis [3]. Focal replacement fibrosis can be detected by cardiovascular magnetic resonance (CMR) with late gadolinium enhancement (LGE) and is observed in up to half of aortic stenosis patients with non-infarct type being the most prevalent [4]. Multicenter trials and meta-analyses have shown that the presence and extent of LGE are associated with higher long-term all-cause and cardiovascular mortality after aortic valve replacement (AVR), indicating more advanced myocardial injury [4, 5]. Focal myocardial fibrosis has been demonstrated to be irreversible following AVR [6, 7], resulting in incomplete LV recovery and worse clinical outcomes, suggesting suboptimal timing of aortic valve intervention in some patients. On the contrary, diffuse interstitial fibrosis has been reported to be reversible with afterload relief and could be utilized as a potential marker of early LV dysfunction [8, 9]. However, the most recent study showed that regression of diffuse fibrosis may be incomplete in certain patients, leading to persistent LV systolic dysfunction and worse survival [10]. Application of T1 mapping techniques, which measures native and postcontrast T1 relaxation time or extracellular volume fraction (ECV), facilitates non-invasive detection and quantification of interstitial fibrosis with high spatial resolution [11–14]. Several studies in AS patients have reported that native T1 and ECV values correlate with the degree of diffuse myocardial fibrosis and predict cardiovascular events and mortality [9, 10, 15]. Thus, myocardial fibrosis detection by CMR is potentially useful for improving patient risk stratification and perhaps can justify earlier aortic valve intervention, before extensive fibrosis and irreversible myocardial damage develop.

Furthermore, because the subendocardial layer is the first to be affected in AS, the longitudinal alignment of myocardial fibers in this layer causes decreased longitudinal contraction, an early sign of LV dysfunction. There are data showing, that global longitudinal strain (GLS) correlate with LV myocardial fibrosis and is a predictor of adverse events in patients with severe AS [16, 17].

Novel diagnostic strategies and more accurate evaluation of the disease severity and consequences of AS are needed in the assessment of subclinical myocardial dysfunction and the detection of myocardial fibrosis to challenge current recommendations for the timing of AVR. To date there are limited data on simultaneous assessment of diffuse myocardial fibrosis by noninvasive multimodality imaging and histological confirmation in severe AS. Optimal T1 mapping application also remains unclear, and data assessing its prognostic value in severe AS are still lacking. Our multicenter prospective trial aims to: (i) non-invasively assess myocardial fibrosis and validate it against histological data in patients undergoing surgical AVR and (ii) assess the impact of myocardial fibrosis on clinical outcomes following AVR in both surgical and TAVR cohorts.

### Hypothesis

Primary hypothesis: diffuse and focal myocardial fibrosis in patients with severe AS is associated with worse immediate (in-hospital) and long-term clinical outcomes, all-cause mortality, and major adverse cardiovascular events (MACEs).

Secondary hypotheses:

- The presence and extent of myocardial fibrosis are associated with markers of LV decompensation.
- The presence and extent of myocardial fibrosis have a negative effect on LV reverse remodeling and patient functional recovery following AVR.

### Study objectives

Primary objective:

- To evaluate the prognostic significance of myocardial fibrosis in patients with severe AS undergoing AVR.

Secondary objectives:

- To identify parameters of multimodality imaging [two-dimensional (2D) echocardiography with an extended myocardial deformation analysis, 1.5 T contrast-enhanced CMR with T1 parametric mapping] predictive of LV decompensation.
- To quantify LV reverse remodeling 12 months following AVR through CMR and echocardiographic measurements.

## Methods

### Study design

**Fig. 1:**
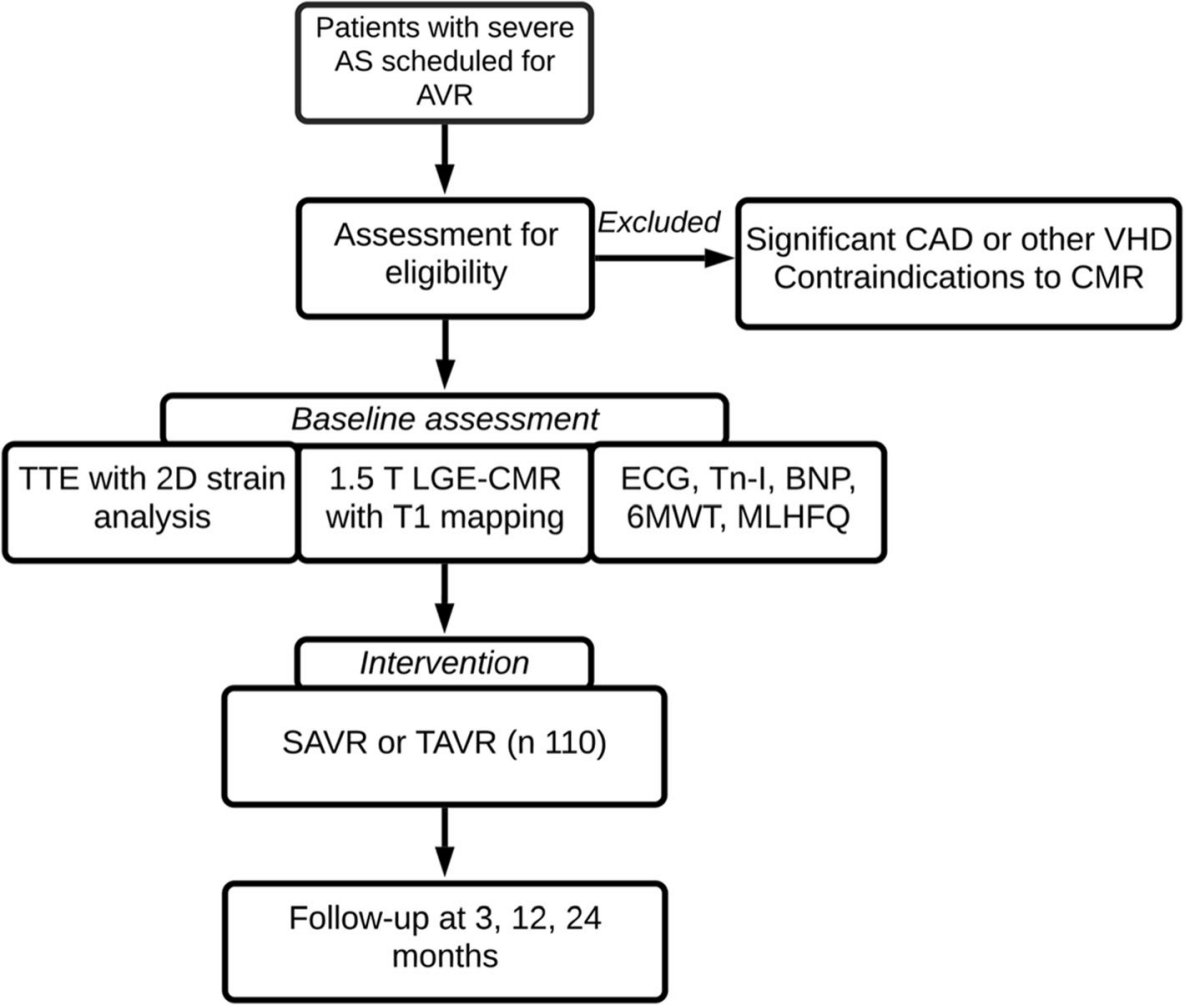
Flow diagram of FIB-AS study. Abbreviations: 6MWT- 6-min walking test, AS- aortic stenosis, BNP- brain natriuretic peptide, CAD- coronary artery disease, LGE-CMR- late gadolinium enhancement cardiovascular magnetic resonance, MLHFQ- Minnesota Living with Heart Failure Questionnaire, SAVR- surgical aortic valve replacement, VHD- valvular heart disease, TAVR- transcatheter aortic valve replacement, Tn-I- troponin I

FIB-AS is a prospective, observational, cohort study with clinical endpoints, conducted in two participating sites (in Lithuania and Denmark) from tertiary care hospitals. A total of 110 patients with severe AS undergoing AVR will be recruited from both institutions, and their data will be collected in a dedicated online database, REDCap (Research Electronic Data Capture) [18]. The choice of aortic valve intervention, either surgical or transcatheter, will be based on the heart team’s decision in accordance to current guidelines [19]. Standard work-up examinations for surgical AVR or TAVR will be conducted, consisting of coronary angiography, echocardiography, computed tomography, electrocardiography, and blood tests. Once consent is confirmed, and prior to the AVR procedure (window of 0–30 days), a contrast-enhanced CMR scan with T1 mapping will be performed, in addition to the standard-of-care. A follow-up CMR scan will be performed 1 year following AVR. Those undergoing surgical AVR will also have a myocardial biopsy sampled at the time of a surgery, which will be sent for histological evaluation. Outcome assessments will continue for a total of 2 years post-intervention. The study flow diagram is presented in Fig. 1**.** Both sites have received approval from local ethics committees (Approval Numbers: for Vilnius University Hospital (VUH): 158200–18/9–1014-558; for Aalborg University Hospital (AAUH): N-20180081. All patients will give written informed consent.

### Study population

We will recruit patients with severe AS requiring AVR according to current treatment recommendations [19]. We will exclude patients with obstructive coronary artery disease requiring revascularization or other significant valvular pathology. The inclusion and exclusion criteria are set in Table 1**.** The recruitment period will be 24 months, from May 2019 to May 2021. We expect to enroll a total of 110 patients: 60 patients undergoing surgical AVR and 50 patients undergoing TAVR.

**Table 1:** Inclusion and exclusion criteria

Inclusion Criteria:	
• Severe AS (defined as AVA ≤1 cm^2^ or AVA index (iAVA) ≤0.6 cm^2^/m^2^ as determined by ultrasound examination)	
• Males and females of any ethnic group ≥18 years of age	
• Signed an informed patient consent form	
Exclusion Criteria:	
• Unable to provide informed consent	
• Severe valvular heart disease other than AS	
• Coronary artery disease requiring revascularization	
• History of myocardial infarction	
• Prior cardiac surgery	
• Severe renal impairment - eGFR < 30 ml/min/1.73 m^2^	
• Any absolute contraindication to CMR	
• Permanent atrial fibrillation	
• Patient with implanted cardiac devices (pacemaker, ICD)	
• Inherited or acquired cardiomyopathy	
• Other medical conditions that limit life expectancy or preclude AVR	
• Pregnant or nursing women	
• Mental condition rendering the patient unable to understand the nature, scope, and possible consequences of the study or to follow the protocol	

Abbreviations: AS- aortic stenosis, AVA- aortic valve area, iAVA- aortic valve area index, AVR- aortic valve replacement, CMR- cardiovascular magnetic resonance, eGFR- estimated glomerular filtration rate, ICD- implantable cardioverter defibrillator

### Aortic valve replacement

Surgical AVR will be performed by standard midline sternotomy or J mini sternotomy with cardiopulmonary bypass and mild hypothermia. The St. Jude Medical Trifecta aortic bioprosthesis (St. Jude Medical, Inc., St. Paul, MN, USA) or mechanical CarboMedics Standard Aortic Valve (CarboMedics, Inc.; Austin, Tex) prostheses of varying sizes will be used according to the surgical team’s or patient’s preference. TAVR will be performed under conscious sedation. A balloon-expandable Edwards Sapien-3 Ultra Valve system, size 23, 26, or 29 mm (Edwards Lifesciences Inc., Irvine, CA, USA), will be deployed. Preferably a transfemoral route of access will be used, when vascular access will be judged suitable.

### Data collection

The risk of interventional mortality will be assessed by calculating European System for Cardiac Operative Risk Evaluation II (EuroSCORE II) risk scores and Society of Thoracic Surgeons (STS) risk scores using the online calculators available at http://www.euroscore.org/calc.html [20] and at http://riskcalc.sts.org/stswebriskcalc [21]. Although not designed for TAVR, the STS score has been validated and proved as a sensitive predictor of 30-day mortality and may be used in both SAVR and TAVR groups [22]. New York Heart Association (NYHA) functional class and 6-min walking test (6MWT) will be applied to assess patients’ functional capacity and performed using standard method [23]. The Minnesota Living with Heart Failure Questionnaire (MLHFQ) [24] will be used to assess health-related quality of life from the patient’s perspective, as it has been specifically validated in patients with aortic valve disease [25].

### Blood testing

Blood samples will be collected and levels of cardiac biomarkers (B-type natriuretic peptide and troponin I) will be tested at baseline and at each follow-up visit. In addition, blood samples will be taken on the day of CMR scanning to determine hematocrit and creatinine concentration.

### Echocardiography

Comprehensive transthoracic 2D echocardiography will be performed using a commercially available Vivid ultrasound (S70, E9 or E95) systems (GE Healthcare, Horten, Norway) and stored on a dedicated workstation for subsequent off-line analysis. AS severity, LV systolic and diastolic functions will be evaluated in accordance with the echocardiographic guidelines [26–28]. Simpson’s biplane method will be used to calculate LV ejection fraction. The aortic valve area at the pre-interventional assessment and the effective prosthetic orifice area in the post-interventional assessment will be calculated using the continuity eq.

#### 2D speckle tracking echocardiography (STE)

From the 2D grey-scale images of the apical two-, three-, and four-chamber views, left ventricular global longitudinal strain (GLS) will be measured and processed offline using commercially available software (EchoPac 112.0.1, GE Medical Systems; Horten, Norway) [29]. The frame rate will be adjusted to 50 to 80 frames/s. The end-systole will be defined by detecting the closure click on the spectral tracing of the pulsed-wave Doppler of aortic valve flow. During analysis, the endocardial border will be traced manually at an end-systolic frame, and the software will automatically trace a region of interest that includes the entire myocardium. The change in length versus the initial length of the speckle pattern over the cardiac cycle will be used to calculate longitudinal strain, with myocardial lengthening or stretching represented as positive strain and myocardial shortening defined as negative strain. GLS will be acquired by the average regional strain curves (16-segment model for 2D STE). 2D STE analysis will be performed only in patients from VUH by an accredited investigator (G.B.). Segments with poor quality tracking or aberrant curves despite manual adjustment will be removed from analysis.

### CMR protocol

#### Basic description

For each patient, baseline and 1-year post-interventional CMR scans will be performed using standard protocols on a 1.5 T Siemens Aera scanner (at VUH) or a 1.5 T GE Discovery MR 450 scanner (at AAUH) with surface coils and prospective ECG triggering [3, 9]. Both sites will use corresponding CMR protocols. LV end-systolic and end-diastolic diameters, as well as maximal (end-diastolic) wall thickness, will be traced and recorded from the short-axis and long-axis views of the standard ECG-gated steady state-free precession cine sequence (8-mm slice thickness). LV volumes, mass, and ejection fraction will be measured using commercially available software (suiteHEART®, NeoSoft, USA or cmr42, Circle Cardiovascular Imaging Inc., Canada) from a stack of sequential 8-mm short axis slices (0–2-mm gap) from the atrio-ventricular ring to the apex. Measurements will be indexed to body surface area in m^2^ (using the DuBois and DuBois formula).

#### LGE imaging

For detection of delayed hyperenhancement, images will be acquired 10–15 min after intravenous administration of Gadobutrol (0.2 mmol/kg) (Gadovist; Bayer AG, Germany) or Gadoteridol (0.2 mmol/kg) (Prohance; Bracco Imaging, Sweden) using a breath-hold segmented inversion recovery fast gradient echo sequence in the short-axis and long-axis planes of the LV, with an 8-mm slice thickness and 20% distance factor. The image parameters (VUH) are: repetition time of 700 ms, echo time 1.42 ms, flip angle 45°, matrix 256 × 184, and field of view 360 mm. For AAUH, the typical imaging parameters are as follows: repetition time 5.79 ms, echo time 2.72 ms, flip angle 15°, matrix 256 × 256, and field of view 380 mm. The optimal inversion time will be optimized for each patient to null normal myocardial signal, ranging from 220 to 320 ms (usually around 240 ms). The region of myocardial fibrosis will be defined as the sum of pixels with signal intensity above 5 standard deviations of the normal remote myocardium in each short-axis slice. The presence of myocardial fibrosis will be qualitatively determined by two independent readers, blinded to clinical data (2 radiologists with > 10 years of experience at VUH- N.V. and D.P., and 2 cardiologists with 8–10 years of experience at AAUH- P.S. and T.Z.).

#### T1 mapping

The present study describes image acquisition parameters by focusing on T1 mapping images, which will be acquired in four-chamber long-axis and short-axis (at the basal and midventricular levels) images before and at 15 min after contrast administration. All T1 mapping images have been acquired using modified Look-Locker inversion-recovery (MOLLI) [30, 31] using the Motion Correction technique (on the Siemens scanner). The following readout parameters will be used: slice thickness: 8 mm; flip angle: 35; field of view: 384 × 327; effective TI: 193 ms; voxel size: 1.5 × 1.5 × 8 mm; TR/TE: 312.64/1.33 ms; partial Fourier: 7/8; and parallel imaging factor: 2 (VUH) and slice thickness 8 mm, flip angle 35, matrix size 256 × 256, inversion time 130 ms, and FOV 380 mm (AAUH).

#### Measurement of the ECV and native T1 values

**Fig. 2:**
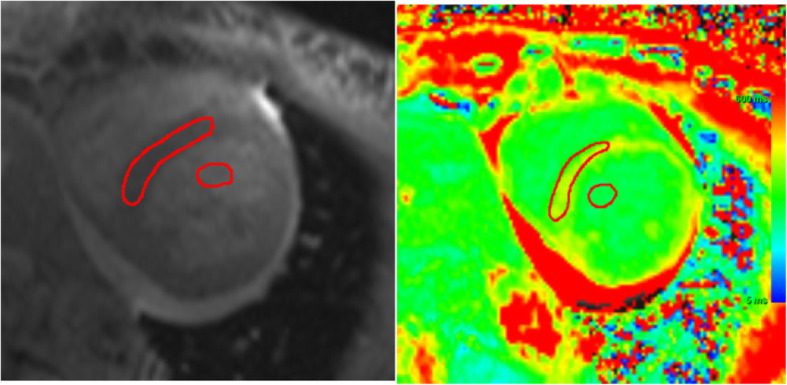
CMR T1 assessment. To measure the T1 value, regions of interest will be drawn manually in the blood and septum at the midventricular level on the short-axis, avoiding papillary muscle

The T1 maps will be generated from the CMR workstation after in-line motion correction just after image acquisition (Siemens scanner) or in post-processing software (GE scanner). Two blinded reviewers will independently review all T1 mapping sequences. Regions of interest will be drawn manually in the blood and septum at the midventricular level on the short-axis image, excluding the myocardium with LGE for T1 measurements [13, 32]. Regions of interest in the septum are chosen for myocardial T1, because this area corresponds to the site of myocardial biopsy. The regions of interest will be drawn on the compact myocardium, and the border of the myocardium will not be included. To measure the T1 value of blood, circular regions of interest will be positioned in the LV cavity, avoiding papillary muscle **(**Fig. 2**).**

**Fig. 3:**
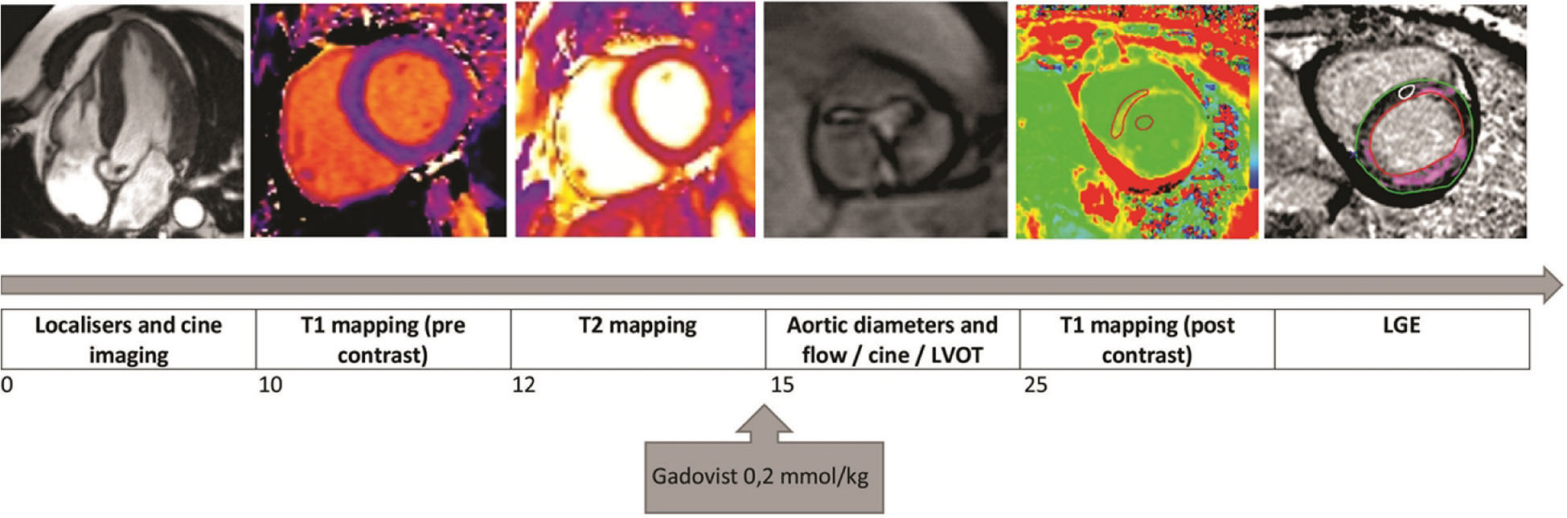
CMR protocol for FIB-AS study

The ECV of the myocardium will be calculated as follows: ECV% = (ΔR1m/ΔR1b) x (1 - hematocrit level) × 100, where R1 is 1/T1, R1m is R1 in the myocardium, R1b is R1 in the blood, and ΔR1 is the change in relaxivity. The change in relaxivity (ΔR1) was determined using the following equation: ΔR1 = R1post - R1pre, where R1post and R1pre are the R1 after and before gadolinium chelate administration, respectively [33]. Blood samples will be taken on the day of CMR scanning to determine hematocrit and creatinine concentration. Interobserver agreement between 2 different experienced observers will be assessed on different days for 10 randomly selected patients. Figure 3 summarizes the CMR sequences used in this study.

### Histological analysis

In patients undergoing surgical AVR biopsy specimens will be obtained under direct vision by the surgical team using a surgical scalpel from the basal anteroseptum just after removal of the diseased aortic valve. All myocardium tissue samples will be fixed in 10% neutral buffered formalin and embedded in paraffin. Sections of 3-μm thickness will be made on a Leica RM2145 microtome and stained by hematoxylin and eosin, Masson‘s trichrome, and Congo red methods. Digital images will be captured by using the Aperio Scan-Scope XT Slide Scanner (Aperio Technologies, Vista, CA, USA) under 20× objective magnification (0.5-μm resolution). Histological slides (typically measuring 20–30 sq./mm) will be processed for evaluation of myocardial fibrosis. An experienced (> 25 years of experience) histologist (E.Ž.) blinded to the clinical and CMR data will investigate all biopsy specimens. The fraction of myocardial volume occupied by collagen tissue (collagen volume fraction) will be determined by quantitative morphometry with an automated image analysis system (HALO™) in sections stained with Masson’s trichrome. Quantification of the myocardial fibrosis area will be performed using HALO™ Classifier Module and HALO™ Area Quantification v1.0 algorithms (IndicaLabs, NM, USA) within manually selected regions of interest enclosing the tissue section and excluding endocardium from the analysis. All study assessments are summarized in Table 2**.**

**Table 2:** Study assessments

Demographic data and comorbidities	
STS risk score	
EuroSCORE II	
Serum biomarkers:	
▸ Brain natriuretic peptide	
▸ Troponin I	
Cardiovascular imaging biomarkers:	
▸ Transthoracic 2D echocardiogram with strain analysis (GLS)	
▸ 1.5 T contrast-enhanced CMR with T1 mapping (Native T1, ECV)	
Myocardial histological analysis:	
▸Quantitative myocardial fibrosis assessment (CVF)	
Functional status and quality of life assessment:	
▸New York Heart Association functional class	
▸Minnesota Living with Heart Failure Questionnaire	
▸6-min walking test	

Abbreviations: 2D- two-dimensional, ECV- extracellular volume, EuroSCORE- European System for Cardiac Operative Risk Evaluation, CMR- cardiovascular magnetic resonance, CVF- collagen volume fraction, GLS- global longitudinal strain, STS- Society of Thoracic Surgeons

### Outcome measures

Primary outcome measure: a composite of all-cause mortality and MACEs (time frame: from 30 days up to 24 months following AVR).

Secondary outcomes measures:

- In-hospital and 30-day all-cause mortality (time frame: 30 days)
- Length of hospital stay
- Time to the event (death or MACE) (time frame: 24 months)
- Cardiovascular mortality (from 30 days up to 24 months following AVR)

Primary outcome endpoints will be defined according to current guidelines: Valve Academic Research Consortium-2 (VARC-2) definitions [34] and guidelines for reporting mortality and morbidity after cardiac valve interventions [35]. Cardiovascular mortality defined as: (1) death due to proximate cardiac cause (e.g., myocardial infarction, cardiac tamponade, worsening heart failure, and low cardiac output syndrome); (2) death caused by non-coronary vascular conditions (e.g., pulmonary embolism, stroke, aortic rupture, or vascular dissection); (3) all procedure-related deaths; (4) all valve-related deaths; and (5) sudden or unwitnessed death [35]. All-cause mortality defined as the sum of cardiovascular and non-cardiovascular deaths, with the latter defined as any death in which the primary cause is clearly related to other than cardiovascular condition. 30-day mortality defined as death occurring within 30 days or during index procedure hospitalization, if the postoperative length of stay is longer than 30 days.

### Clinical follow-up

**Fig. 4:**
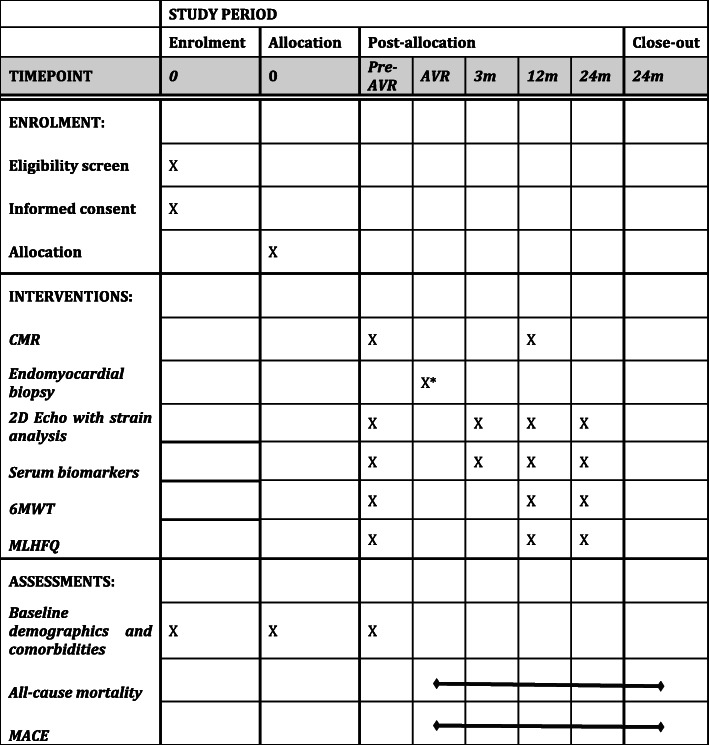
Standard protocol items: recommendation for interventional trials (SPIRIT) figure of participant timeline. Abbreviations: 2D- two-dimensional, 6MWT- 6-min walking test, CMR- cardiovascular magnetic resonance, MACEs- major adverse cardiovascular events, m- months, MLHFQ- Minnesota Living with Heart Failure Questionnaire. *Endomyocardial biopsy will be performed only in patients undergoing surgical AVR

Patients will be routinely followed and managed according to available guidelines. Clinical outcome data will be collected from patient clinical visits at 3, 12, and 24 months following AVR. Serum biomarker analysis and 2D echocardiography with STE analysis will be performed at each follow-up visit. A second CMR scan will be performed 1 year after AVR. The 6MWT will be performed, and the MLHF questionnaire will be administered at the 12-month follow-up visit. FIB-AS study follows the standard protocol items: recommendation for interventional trials (SPIRIT) guidelines [36]. The study timeline is presented in Fig. 4**.**

### Statistical analysis

- Linear regression analysis to model the relationship between the extent of myocardial fibrosis and patient outcomes.
- Univariate and multivariate regression analysis of CMR-derived predictors of adverse clinical events.
- Comparison of clinical, serum, and imaging (CMR and echocardiography) biomarker data between patients with different extents of fibrosis.

The statistical analysis will be performed under the supervision of V.S. at the Institute of Applied Mathematics, Vilnius University Faculty of Mathematics and Informatics. Descriptive statistics will be used to summarize the demographic, clinical, and imaging data characteristics of each patient. Categorical variables will be presented as the frequency and percentage, and continuous variables will be expressed as the mean and standard deviation or median and interquartile range. The 95% confidence interval will be reported for primary outcomes. Patients will be grouped, depending on the presence and extent of histologically measured myocardial fibrosis, and their clinical and imaging data will be compared for significant differences. Bivariate correlation analysis of the native T1, ECV, and GLS values with the degree of histologically measured myocardial fibrosis will be performed. Pearson or Spearman correlation coefficient will be used as the measure of correlation. To identify independent predictors of the degree of myocardial fibrosis, multivariate stepwise regression analysis will be performed separately for native T1, ECV, and GLS values, along with demographic and clinical data. The composite event will be assessed from a simple Cox model for survival analysis using ECV, native T1, and GLS, and their c-indexes will be compared for how well each marker predicts the clinical outcome. In addition, the Kaplan–Meier method will be used for the cumulative survival analysis with log-rank test to assess statistical differences between the curves of patients with different degree of myocardial fibrosis. A two-tailed *p*-value of < 0.05 will be considered statistically significant. The statistical analysis will be performed using the R language and environment for statistical computing (version 3.5.1) [37].

### Sample size justification

A sample size calculation was performed for the primary outcome: a composite of all-cause mortality and MACEs following AVR. To estimate the sample size required for the detection of a hazard ratio for ECV% of 1.32, R package powerSurvEpi was used [38]. Calculations were made assuming that the Cox proportional hazards regression model applies and taking into consideration a correlation of ECV% with other possible confounding covariates. On the basis of previous studies [15, 17] and assuming that the power of the test should be at least 0.8, a sample size of 100 patients is required. After an adjustment for 10% patient loss or withdrawal, a total sample size of 110 patients was selected. The selected sample size is comparable to other studies testing the association of myocardial fibrosis with adverse clinical outcomes.

### Study timetable

The ethics application was approved in March 2018. Study enrollment started in May 2019, and recruitment is expected to be completed in May 2021, with a further 24 months for follow-up, post-processing, and close-out of the study. The main study paper will be submitted within 6 months of the study close-out.

### Dissemination of results

The study results will be presented to the participating physicians and the general medical community. Following the complete data collection, the manuscript(s) based on the trial results will be submitted to peer-reviewed journals. Authorship criteria as defined by the International Committee of Medical Journal Editors will be followed.

## Discussion

FIB-AS is a study intended to investigate the role of multimodality imaging in the assessment of myocardial fibrosis in severe aortic stenosis patients and its prognostic significance in post-interventional outcomes. We sought to assess diffuse myocardial fibrosis by measuring the ECV and native T1 values obtained from CMR with T1 mapping and GLS from STE and to validate these results against the degree of myocardial fibrosis found from the histological examination. In addition, we will explore LV reverse remodeling following aortic valve intervention and its associations with patient clinical recovery. The results of the study will expand the current knowledge of cardiac remodeling in AS and will bring additional data on myocardial fibrosis and its clinical implications following AVR.

### Strengths

- The present prospective multicenter study is designed to explore associations between left ventricular myocardial fibrosis and clinical outcomes in severe AS patients undergoing AVR. It has clearly established aims, inclusion and exclusion criteria, as well as defined methods and endpoints.
- The non-invasive measurement of myocardial fibrosis by CMR with T1 mapping is validated against invasive histological assessment.
- The trial is restricted to isolated AS, excluding cardiac pathologies which could possibly contribute to myocardial fibrosis burden, such as obstructive coronary heart disease and other significant valvular heart diseases.
- Inclusion of both surgical and TAVR cohorts will allow the investigation of patients with different risk profiles.

### Limitations

- The selected sample size may be inadequate to allow a subgroup analysis.
- Limitations of CMR to establish diffuse and focal fibrosis: no reference regions of normal myocardium due to diffuse fibrosis, arbitrary selection of threshold of signal intensity, overlap of T1 values between normal and diseased myocardium.
- The FIB-AS study excludes patients with comorbidities, such as obstructive coronary artery disease, a history of myocardial infarction, renal failure, and persistent atrial arrhythmias; therefore, our results should not be overgeneralized to a broader AS patient population.

## Data Availability

Not applicable. Study patient enrollment and data collection is currently ongoing and no datasets were generated for analysis yet.

## Abbreviations

2D STE: Two-dimensional speckle tracking echocardiography
6MWT: 6-min walking test
AAUH: Aalborg University hospital
AS: Aortic stenosis
AVR: Aortic valve replacement
CAD: Coronary artery disease
CMR: Cardiovascular magnetic resonance
ECV: Extracellular volume
GLS: Global longitudinal strain
LGE: Late gadolinium enhancement
MOLLI: Modified look-locker inversion-recovery
NYHA: New York heart association
TAVR: Transcatheter aortic valve replacement
VUH: Vilnius University hospital
